# Compensatory evolution for a gene deletion is not limited to its immediate functional network

**DOI:** 10.1186/1471-2148-9-106

**Published:** 2009-05-16

**Authors:** WR Harcombe, R Springman, JJ Bull

**Affiliations:** 1 Section of Integrative Biology, Institute for Cell and Molecular Biology, Center for Computational Biology and Bioinformatics, University of Texas, Austin, Texas 78712, USA

## Abstract

**Background:**

Genetic disruption of an important phenotype should favor compensatory mutations that restore the phenotype. If the genetic basis of the phenotype is modular, with a network of interacting genes whose functions are specific to that phenotype, compensatory mutations are expected among the genes of the affected network. This perspective was tested in the bacteriophage T3 using a genome deleted of its DNA ligase gene, disrupting DNA metabolism.

**Results:**

In two replicate, long-term adaptations, phage compensatory evolution accommodated the low ligase level provided by the host without reinventing its own ligase. In both lines, fitness increased substantially but remained well below that of the intact genome. Each line accumulated over a dozen compensating mutations during long-term adaptation, and as expected, many of the compensatory changes were within the DNA metabolism network. However, several compensatory changes were outside the network and defy any role in DNA metabolism or biochemical connection to the disruption. In one line, these extra-network changes were essential to the recovery. The genes experiencing compensatory changes were moderately conserved between T3 and its relative T7 (25% diverged), but the involvement of extra-network changes was greater in T3.

**Conclusion:**

Compensatory evolution was only partly limited to the known functionally interacting partners of the deleted gene. Thus gene interactions contributing to fitness were more extensive than suggested by the functional properties currently ascribed to the genes. Compensatory evolution offers an easy method of discovering genome interactions among specific elements that does not rest on an a priori knowledge of those elements or their interactions.

## Background

Genomics is providing insights to the nature of and interactions among elements within genomes. Furthermore, because the genome provides the platform for evolution, insights from genomics should yield insights to evolution. Information goes the other way as well: evolutionary changes reflect the workings of the genome and so can be used to help understand the genome.

This understanding has motivated diverse approaches to the use of evolution to discover genome interactions. One approach is to compare natural patterns of molecular co-evolution between genes of the same genome: interacting elements are expected to show correlated rates of evolution [1]. Another approach is more direct: engineer a genomic defect, experimentally evolve the genome to higher fitness, and observe the genetic basis of its recovery. The mutations that 'compensate' for the defect should map on the interacting partners of the defective element, in the local network of the affected gene(s). One implementation of this second approach, long used in genetics, is to study mutations that single-handedly rescue a lethal mutation, known as second-site suppressors. A more encompassing version of this evolutionary approach involves an extended experimental adaptation of a defective genome and identification of the full spectrum of compensatory changes. A defective gene may be rescued either by mutations in the elements interacting with it or by mutations that restore the lost function, [2,3] but only the first of these will reveal the interactions.

Here we test that perspective with an engineered deletion of the DNA ligase gene in the bacteriophage T3. This phage encodes at least 6 other proteins with major functions in DNA metabolism: DNA polymerase, endonuclease, exonuclease, ssDNA binding protein, and the overlapping, in-frame helicase and primase [4]. The phage RNA polymerase and an inhibitor of host dGTPase have minor roles in phage DNA metabolism. Gp4.7 may also have a role in DNA metabolism based on homology to a subunit of *E. coli *DNA polymerase [5]. Our expectation is that most compensatory changes in response to ligase deletion, and especially the important ones, will reside in the DNA metabolism network. Ligase has no known physical interactions with other DNA metabolism proteins, so effects of its absence on the network are largely functional. Yet, quantitative knowledge of these functional interactions is primitive and does not readily assist in predicting which genes will be most affected by the deletion of ligase, except perhaps endonuclease [6].

The study here parallels one of T7 [7], a relative of T3. Both genomes are approximately 40 kb dsDNA with a common set of essential genes and most non-essential genes in the same gene order; nucleotide divergence between them is about 25% [4,8]. Compensatory evolution in T7 deleted for its ligase gene resided largely within the DNA metabolism network, although some changes were in genes or elements of unknown function. Thus replication of the study with T3 tests the robustness of the 'network' model of compensatory evolution and also reveals whether the locations of compensatory changes are conserved.

## Results

**Figure 1:**
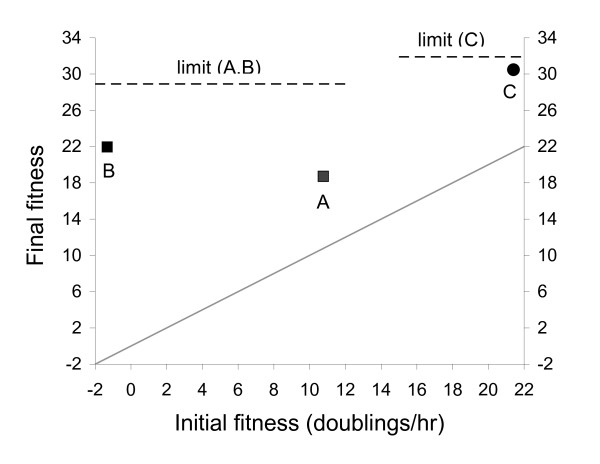
**Fitness evolution of T3 deleted for the ligase gene and adapted to the ligase-defective host (points A, B) or to the ligase-normal host (C)**. Final fitness equals initial fitness for the oblique line, thus the vertical distance from the oblique line to the labeled data point represents the total improvement during adaptation. Adaptation of A was initiated from T3Δ1.3A_0_, adaptations of B and C from T3Δ1.3B_0_. Initial fitness of T3Δ1.3B_0 _was negative on the ligase-defective host, so the phage could not initially maintain its numbers and had to be propagated initially on plates for the B adaptation. In contrast, initial fitness of T3Δ1.3A_0 _on the ligase normal host was over 20 doublings/hr. Initial fitness of T3Δ1.3A_0 _was higher, due to the acquisition of 5 changes by the first step at which the isolate could be identified as carrying the deletion. Final fitnesses of all three were substantially improved over initial. However, final fitnesses of A and B fell short of the fitness limit (28.9) of the ligase+ T3 adapted to the same host [designated limit (A, B)]. Adaptation of T3Δ1.3B_0 _to the ligase-normal host (point C) resulted in a final fitness approaching the presumed fitness limit. Standard errors in both the vertical and horizontal dimensions are indicated, often obscured by the symbols.

Two lines of T3 deleted for ligase were adapted for rapid growth (designated T3Δ1.3A and T3Δ1.3B; subscript 0 will indicate the initial phage, subscript E the evolved, endpoint phage). The host used was itself ligase-defective to magnify the fitness impact of the phage deletion. Final fitnesses of both T3Δ1.3 adaptations improved over initial fitnesses but were still well below that of a control, wild-type T3 adaptation with ligase gene intact (T3^+^_E_, Fig. 1). Initial fitnesses of the two lines differed substantially, the basis of which will be addressed below.

The fitness improvements should reflect underlying genomic changes, but those changes could either be compensatory for the ligase deletion or could be generally beneficial by augmenting growth under the passage conditions. Based on our recombination-based assessment of compensatory evolution (Methods), each line accumulated slightly more than a dozen compensatory mutations, spread across at least 10 genes and other genetic elements (Table 1). In addition, three mutations of general benefit were observed in one line, none in the other, and one mutation of ambiguous compensatory status was observed in each line (Additional files 1, 2). The consensus sequence of T3Δ1.3A_E _also revealed four mutations polymorphic over wild-type that were not near fixation and thus of such possibly weak benefit as to defy resolution in our compensatory assay (Additional file 1).

**Table 1:** Functional locations of strictly compensatory changes for deletion of phage DNA ligase

**Phage element**	**T3Δ1.3 A_E_**	**T3Δ1.3 B_E_**	**T7Δ1.3_E_**
DNA metabolism			
ss DNA binding (*2.5*)	+^1^		
endonuclease (*3*)	+*	+	+
helicase/primase (*4A, B*)	+	+	+
DNA polymerase (*5*)		+	+
Exonuclease (*6*)		+	
Virion			
major capsid (*10*)		+	
tail A (*11*)	+*		+^2^
tail B (*12*)	+		
internal core (*16*)		+*	
tail fiber (*17*)	+*	+	
Other			
protein kinase (*0.7*)	+		
*1.05 *(unknown)	+*	+	A
*1.5 *(unknown)	+	+	+
*1.6 *(unknown)	+		
host RNAP inhibition (*2*)		+	
*2.8 *(unknown)	A	A	+
lysozyme (*3.5*)		+	
*5.3 *(unknown)		+	
RNase III site (*6.5*)	+		+
Ribosomal binding site (*5*)	+		
Packaging (*19*)/*19.2 *(unknown)		+	

+ = a gene that acquired at least one compensatory mutation. A = a gene that is absent from the respective genome. T3 data from Additional files 1 and 2; T7 data are from Rokyta et al. [7]; except for the tail mutation which was from the T7Δ1.3 adaptation done for this study. Omitted are changes that were not strictly compensatory and changes that were polymorphic in the endpoint, evolved populations. Changes in T3Δ1.3 A_E _and T3Δ1.3 B_E _indicated with an asterisk (*) were detected in the initial isolates, prior to exposure to the ligase-host.

^1 ^Consensus sequence was indicative of apparent fixation, but only 1 of 2 isolates carried this change. Designation as compensatory is based on the consensus sequence profile.

^2 ^24559 A->G D111G; observed in a T7Δ*1.3 *adaptation done for this study; compensatory.

Our primary interest is the nature of the strictly compensatory changes – whether they reside within or outside of the DNA metabolism network. Biochemical and other phenotypic effects of mutations are not addressed, just their locations.

### Compensatory evolution in the DNA metabolism network

**Figure 2:**
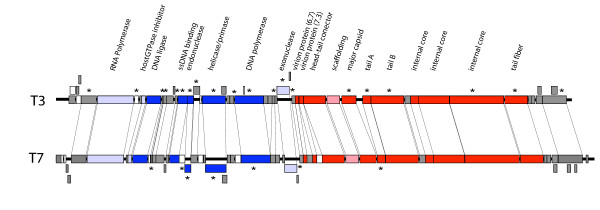
**Genome organization and location of mutations in T3 and T7**. The identities and order of all essential genes and most non-essential genes are the same in both phages. Genes shown in blue function in DNA metabolism (dark blue indicates major roles, light blue lesser roles). Genes in red encode virion proteins (the light red gene is for scaffolding, absent in the mature virion). The three internal core genes have unique functions and identities. Genes shown in gray have other functions or their functions are not known. Asterisks are shown above the genes in which compensatory substitutions were observed in either T3 adaptation and shown below the genes of T7 that experienced compensatory evolution. Overlapping genes are offset (the overlap of helicase and primase and the overlap of the two forms of the capsid gene are each shown as a single gene).

**Figure 3:**
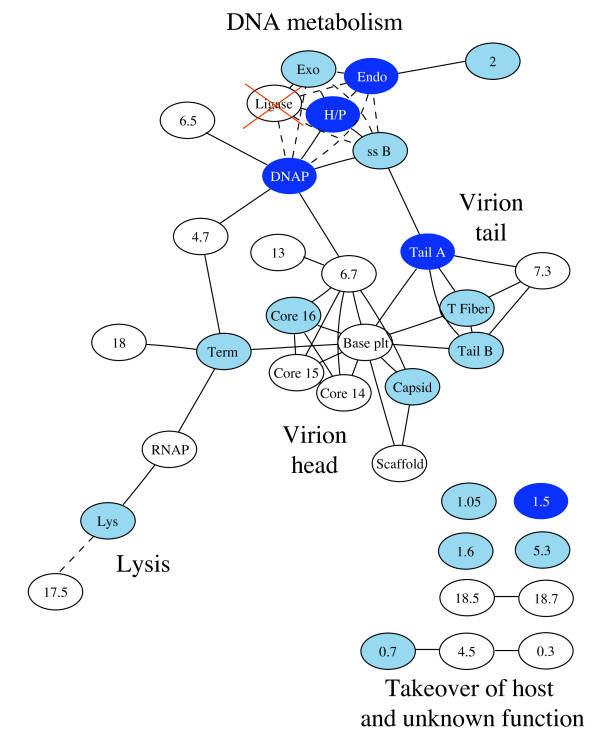
**Genome network of phages T3 and T7**. Genes (ovals) are identified with partial names or numbers; the only gene shown that is not found in both phages is *1.05*, found only in T3. Light blue genes are those that evolved compensatory changes for ligase deletion in either T7 or T3; dark blue genes evolved compensatory changes in both T7 and at least one line of T3. Solid lines indicate direct contacts known from biochemistry, contacts inferred from yeast-2-hybrid data, or contacts inferred from known associations. For example, the three core proteins are found inside the phage head, and it is not known which of them contact each other and which contact other head proteins. Dashed lines indicate known functional interactions for two discrete phenotypes (DNA metabolism, lysis). The phage RNAP obviously interacts functionally with most of these genes through its expression of them, and those interactions are not shown. Many non-essential genes are omitted; the few listed with no connections are non-essential under lab growth conditions but evolved compensatory changes. This network represents the state of knowledge for T7 (and thus T3), but the T7 network has not been extensively explored, so this network should be acknowledged as incomplete. Furthermore, there is yet no structure of the T3 or T7 virion that reveals the locations of gp6.7, gp7.3, or gp13 nor of the relative locations of tail A to tail B, so many of the connections shown here for those proteins have been assigned by relatively weak inference. Sources include [5,8-14]. Gene numbers and functions are given in Additional file 1. Abbreviations (gene name, number): Lys (lysozyme, *3.5*), Holin (*17.5*), RNAP (RNA polymerase, *1*), DNAP (DNA polymerase, *5*), Tnase (terminase, *19*), Scaffold (*9*), Capsid (major/minor capsid, *10A*, *10B*), T Fiber (tail fiber, *17*), Tail A (*11*), Tail B (*12*), Endo (endonuclease, *3*), Exo (exonuclease, *6*), H/P (helicase/primase, *4A, 4B*), ssB (ss DNA binding protein, *2.5*).

Both adapted T3Δ1.3 lines experienced compensatory changes in 3–4 genes of the DNA metabolism network (Table 1; Fig. 2 gives the locations of the mutations on the linear genome; Fig. 3 gives them in the context of current network information [5,8-14]). Endonuclease and the overlapping helicase/primase genes were the only ones compensating in both T3 adaptations, but three other DNA metabolism genes were involved in either of the adaptations. When the same gene experienced compensatory changes in both adaptations, the protein residues affected were not the same (Additional file 1, 2).

### Compensatory evolution in virion proteins

The T3 adaptations evolved several compensatory changes outside of DNA metabolism (Table 1, Fig. 2, Fig. 3). Several changes occurred in genes encoding virion proteins with no known connection to DNA metabolism. Furthermore, the virion genes evolving in T3Δ1.3A_E _tended to differ from those in T3Δ1.3B_E_. T3Δ1.3A_E _was the more fully characterized, largely because it exhibited a surprising early evolution of changes.

T3Δ1.3A_0 _had acquired five mutations before being exposed to the ligase-defective host (Additional file 1). Isolation of a deletion mutant involved 3 platings on a ligase-normal host before exposure to the ligase-defective host, during which time these mutations appeared (see Methods). Three of them resided in tail and tail fiber genes, whereas only one was in a gene involved in DNA metabolism (endonuclease, gene *3*). The fifth change was a deletion in a gene of unknown function (*1.05*). One implication of this early evolution is that the A and B lines were no longer identical when first exposed to the ligase-defective host.

Surprisingly, these early tail and tail fiber changes were compensatory for the ligase deletion. Along with one other tail mutation that evolved later, they were even found to be collectively essential to the viability of the adapted T3Δ1.3A_E_. Specifically, a recombinant phage was constructed with its left end from the evolved T3Δ1.3A_E _and the right end from the pre-adapted T3^+^_E _(the restriction site used for this exchange was in gene *7.3*, between the DNA metabolism genes and structural genes of the virion). This phage lacked all tail and tail fiber changes from the adaptation but retained all other changes from T3Δ1.3A_E_. It could not form plaques on the ligase-defective host but could plaque on the ligase-normal host (data not shown). Why those tail and tail fiber changes should be essential on the ligase-defective host in a genome with several other compensatory changes is not clear, but the result indicates strong interactions among the compensatory changes.

The T3Δ1.3B adaptation differed from the T3Δ1.3A adaptation in three ways. First, T3Δ1.3B_0 _had low fitness, less than zero. Second, and undoubtedly related to the first point, T3Δ1.3B_0 _acquired only one mutation during its isolation (in an internal core protein gene). Third, T3Δ1.3B_E _did not acquire any tail changes, although it did acquire one compensatory tail fiber change. (The tail is considered distinct from the tail fiber.) In fact, outside of DNA metabolism, there was little overlap in the suite of genes that acquired compensatory mutations in T3Δ1.3B_E _and T3Δ1.3A_E_.

### Other compensatory evolution

In the T3 lines, some compensatory changes were in genes or elements with functions either outside both DNA metabolism and virion components or with unknown functions (Table 1). Some changes can be interpreted as possibly regulatory, such as the substitution in the RNAse III site upstream of *6.5 *and the change in lysozyme [one function of lysozyme is to bind the phage RNA polymerase and shift its specificity in favor of late genes; [15]]. Major and presumably destructive changes to gene *1.5 *were common to both T3 adaptations (a deletion and a nonsense mutation).

### Evolution on a ligase-normal host

The T3Δ1.3B_0 _phage was adapted to the ligase-normal host (Fig. 1, label C). A profound effect of host ligase is evident from the fact that T3Δ1.3B_0 _had a fitness of 24 doublings/hr higher on the ligase-normal host than on the ligase-defective host. Nonetheless, T3Δ1.3B_0 _fitness on the ligase-normal host improved by nearly 8 doublings/hr after adaptation. Its genome was sequenced only selectively – over all DNA metabolism genes and over some of the virion protein genes. Surprisingly, the only change observed was in gene *6.7 *(the same change observed in B_E_), a gene whose only known role is in adsorption. Adaptation of T3Δ1.3A_0 _to the ligase-normal host was not attempted (it carried too many compensatory mutations).

### T7 revisited

Tail changes were not reported for the adaptation of T7Δ1.3 [7], but the involvement of essential compensatory tail changes in the T3Δ1.3A line led us to reconsider that outcome (Table 1). The long-term T7Δ1.3 line from Rokyta *et al*. carried a change in tail B, but this mutation had arisen prior to the deletion and thus was not strictly compensatory (that study did not report mutations found in the pre-adapted phage). Here, a short-term adaptation was conducted with an independently created T7Δ1.3 genome. That line evolved a compensatory change in the tail A gene (only the tail genes were sequenced in this phage). Thus involvement of virion proteins in compensatory evolution for a ligase deletion appears common to both T3 and T7.

## Discussion

Since the discovery of genes as discrete, physical units, one of the cornerstones of genetics has been that genes have distinct functions and thus functionally interact with limited sets of other genes. With the advent of genomics methods, this understanding has culminated in representations of genomes as connected networks of interacting parts [e.g., [16,17]]. As our understanding of genomes improves, one truly exciting challenge is to discover how evolution maps onto the network and shapes it [18,19].

We tested a simple model of the relationship between genome interactions and evolution: a defect within a network of interacting genes should favor compensating mutations in other genes of the network. Bacteriophage T3 with a crippling deletion of the ligase gene was adapted to see whether compensatory changes would occur primarily in the phage's other DNA metabolism genes. Phage DNA ligase is not essential in the presence of normal cellular ligase, but we used a host with impaired ligase, thus rendering phage ligase essential (except when offset compensating mutations). Ligase is not thought to physically contact the other genes in its network, merely interacting with them functionally to replicate DNA. Phage DNA replication involves promiscuous pairing of strands between different molecules, and resolution of the consequent Holiday junctions involves cutting the DNA (with phage endonuclease) and subsequent ligation of the ends. Thus the functional interaction of ligase and endonuclease is strong [6], but reduction of ligase activity is also expected to impact other processes in DNA metabolism, at least indirectly.

There are two ways that a metabolic network can recover from the loss of a component: re-establish the function of the component or compensate for the loss of the function. Several previous studies experimental studies of evolution in response to knockouts have observed re-establishment of function; examples include studies on networks involved in lactose metabolism in *E. coli *[1,2], lysis in phage [20] and phage host range [21,22]. Here, re-establishment was unlikely because we removed a gene with unique activity. As a result we were able to investigate how a genome compensates for the loss of a function by altering other steps in a metabolic pathway [23-25]. Quantitative estimates of biochemical parameters do not exist for any of the steps in T7 DNA metabolism, so the predictions and observations here address only qualitative properties of the compensatory evolution, i.e., the genes experiencing compensatory evolution.

Two lines of T3 deleted for the ligase gene were adapted, fitness improved, and approximately a dozen compensatory substitutions were observed in each line. Compensatory mutations in both lines included the DNA metabolism network (Table 1, Figs. 2, 3). All six remaining DNA metabolism genes acquired compensatory mutations in one line or the other; however, only three of the six genes acquired mutations in both lines, and even when the same genes exhibited compensatory evolution in both lines, different protein residues were affected. Thus, involvement of the DNA metabolism network appears to be more consistent than involvement of individual genes or of specific protein residues. Functional impacts of those mutations are not known, but parallel evolution at the gene level is most easily explained if it has similar phenotypic effects despite the lack of parallelism in the residues affected.

Compensatory substitutions also occurred outside the DNA metabolism network (Figs. 2, 3). Common to both lines was an involvement of virion protein genes, the tail and tail fiber in one line, and the tail fiber, major capsid protein and an internal core protein in the other. Furthermore, at least some of these extra-network changes had major fitness effects: tail changes were essential to viability in one line.

An earlier long-term adaptation of T7 carrying a ligase deletion parallels the T3 results in some interesting ways [[7]; changes listed in our Table 2]. Foremost was the involvement of DNA metabolism genes in T7. The three DNA metabolism genes that acquired compensatory mutations in both T3 adaptations (endonuclease, helicase, and primase) also acquired compensatory mutations in T7. The importance of endonuclease changes in compensating for a ligase defect was anticipated by Sadowski [6], who noted that ligase and endonuclease provide opposing functions and thus need to be balanced with each other. Thus changes in the level of one should be compensated by corresponding changes in the level of the other. There is no similar justification for anticipating the invariant compensatory evolution of helicase or primase any more than of other DNA metabolism genes, however.

**Table 2:** Sequence differences between pre-adapted T3 (T3^+^_E_) and the published wild-type T3^+^

Nucleotide position	Change	Gene
9606	CG insert	endonuclease (*3*)
9625	G insert	endonuclease (*3*)
9968	G insert	endonuclease (*3*)
9973	G deletion	endonuclease (*3*)
19676-7	CA->AC	head-tail connector (*8*)
25203P	A->G	tail B (*12*)
37621P	G->A	unknown (*19.5*)
37630P	A->G	unknown (*19.5*)

Sequences were determined as a consensus of the phage population and compared to the published sequence of T3 [4]; the relative abundance of a base had to exceed 10% of the total before it was considered real.

P – polymorphic

More interesting, perhaps, was the parallel evolution in T7 and T3 of compensatory mutations outside of the DNA metabolism network. First, mutations that knocked out gene *1.5 *appeared in T7 and both lines of T3. Gene *1.5 *is a short protein (less than 30 AA) whose function is unknown. The protein has 33% similarity between T3 and T7 and hence is far less conserved than are most other genes common to these phages. Despite this lack of sequence conservation, the parallel loss points to a conserved function, likely in a network (DNA metabolism) that is more conserved than the protein itself. Second, a substitution in the RNAse III site upstream of *6.5 *appeared in T7 and in one line of T3. This mutation points to common regulatory responses in the two phages, which is not surprising given their similarities in many regulatory elements. Last, all adaptations acquired mutations in some component of the tail (either tail or tail fiber). In one of two adaptations, T7 evolved a compensatory change in a tail gene. Thus, despite approximately 25% sequence divergence between T7 and T3, some striking parallels exist in the evolutionary responses to ligase deletion, even in elements that are not known to be involved in DNA metabolism.

It is difficult to explain why genes outside the recognized DNA metabolism network were involved in compensatory evolution of the ligase deletion. One obvious possibility is that these genes actually do physically interact with ligase or other proteins involved in DNA metabolism, but the interactions have escaped detection. For example, a systems analysis of DNA damage response pathways in yeast revealed the involvement of many more genes than had been identified by less comprehensive methods [26]. Furthermore, data on neofunctionalization of gene duplicates suggest that genes may often have cryptic secondary functions [27]. To address the possibility of cryptic interactions, a T3 was constructed to carry the T7 ligase gene instead of its own (experiment not described above). If ligase was involved in unknown interactions, this exchange might be expected to lower fitness and select changes in the interacting genes, especially as the two proteins are only 67% similar. The exchange had only a small effect on fitness and only slight compensatory evolution was observed (fitnesses were 28.9, 27.5, and 28.2 before the exchange, immediately after the exchange, and after the recovery, respectively). These results do not support a wealth of important, hidden interactions for ligase.

A second explanation for the inclusion of genes outside DNA metabolism is that the severe disruption caused by the ligase deletion impacts the entire phage genome network, and compensatory changes in this broader network are favored to coordinate the life cycle with the retarded DNA replication. If so, lessening the initial impact of the ligase deletion might then be expected to limit the compensatory evolution to the DNA network. Even when grown on a ligase-normal host, the host ligase does not fully compensate for the absent phage ligase, possibly because expression level is optimized for the host, not the phage. Yet, normal host ligase activity should decrease the impact of the ligase deletion. Indeed the impact of the phage ligase deletion was much lower on a ligase-normal host than on a ligase-defective host, and adaptation on the ligase-normal host resulted in improved fitness. However, no compensatory mutations occurred in DNA metabolism. Thus, we have no promising models to account for the observed compensatory mutations outside of DNA metabolism in response to the phage ligase deletion. It is perhaps worth noting that yeast-2-hybrid analyses also lead to many unexpected interactions; however, yeast-2-hybrid analyses are more prone to spurious technical artifacts than the method used here.

This work dovetails with emerging evidence that genomic evolution is often not confined to the local genetic network thought to control the affected phenotypes. In the yeast *S. cervisiae*, adaptation to high ethanol was accomplished through change in a component of global transcription that has no known direct interaction with ethanol tolerance [28]. Genes whose expression changed did not group into any particular functional network. In another study, *E. coli *that were adapted to use glycerol as a carbon source acquired mutations both within and outside the glycerol metabolic pathway [29]. Likewise, many mutations affecting human genetics diseases have defied a priori knowledge [30].

## Conclusion

A genome was engineered to destroy a gene with a single, well-defined function. Experimental evolution was used to study how the genome would compensate, with a particular interest in whether the compensatory changes would occur in the network of partners that functionally interact with the missing gene. Evolution did not recreate the missing function, but instead compensated the loss through changes in other genes. Many of the compensatory changes were among the functional partners of the missing gene. However, many compensatory mutations were not among functional partners, and these have no obvious explanation, even following investigation with subsequent genetic manipulations and adaptations. Along with a few other experimental evolution studies, this study finds that an a priori phenotypic challenge leads to a mix of plausible and enigmatic compensatory evolution. Despite the appearance that genomes are organized into functional networks controlling defined phenotypes [16,17], phenotype evolution often fails to map onto those networks in an obvious way [28,29]. Studies that only look for changes within the candidate network are likely to find mutations, but they are also likely to miss important dimensions of adaptation. Hence, they run the risk of circularity – observing only what is expected.

Our study has illustrated a method for uncovering genomic interactions that is easily applied in an experimental setting, is comparative, and requires no a priori knowledge of interactions (although such knowledge is useful for interpreting the changes). Discoveries made with this method should complement those of other methods [1]. The method uncovers interactions at a functional level and that do not require protein-protein contacts. These types of interactions will likely be important both in evolution and in the design of synthetic genomes of the future.

## Methods

### Phage and Bacteria

The virus used in this study was T3, a double stranded DNA bacteriophage with a genome size of approximately 38 kbp. The reference sequence used here is that of Pajunen *et al*. [4]. Bacterial hosts were derivatives of *Escherichia coli *B: BL21 and IJ434. BL21 has normal host ligase activity (and is referred to as the 'ligase-normal' host). IJ434 carries a defective ligase [31] and will be referred to as the 'ligase-defective' host. IJ434 also carries a mutation (*optA1*) enhancing levels of dGTPase [32], but the lattermutation is not thought to be important for this study. Normal T7 and T3 growth using IJ434 as host requires the activities of genes *1.2 *(anti-dGTPase) and *1.3 *(DNA ligase). The host IJ1126 (*E. coli *K-12 *recB21 recC22 sbcA5 endA gal thi *Su^+ ^(*mcrC-mrr*)*102*::Tn10) was used for transfection of phage genomic DNA; it has normal host ligase activity.

### Phage growth methods and fitness assays

Except for some initial passages noted below, adaptations of phage used serial transfer in liquid at 37°C, using 125 mL flasks with 10 mL of LB media (10 g NaCl, 10 g Bacto tryptone, 5 g Bacto yeast extract per liter) shaken at 200 rpm for aeration. Cells from frozen aliquots (-80°C) were thawed and added to achieve a density of ~10^8 ^bacteria/mL after 1 hr, at which time phage were added. This phage-cell mix was grown long enough to achieve a phage density allowing transfer of at least 10^5 ^phage into a new flask of cells. However, phage titers were often allowed to reach moderate to high density. At times, the cultures were even allowed to lyse before transfer to enhance recombination and avoid clonal interference of otherwise competing beneficial mutations. Transfers of the phage-cell suspension into new flasks were made directly, without chloroform treatment. A sequence of uninterrupted transfers continued for at least one hour (often several hours, across several flasks) before halting the process with chloroform and creating a stock to be used when continuing adaptation at a later time. Although many phages have latent periods (lysis times) approaching an hour, that of T3 is approximately 20 minutes [IJ Molineux, pers. communication]. Thus an hour of continuous growth allows the timing of infections to become asynchronous, approaching a 'stable age' distribution suitable for fitness measurements of exponential growth (see below as well).

This study focuses primarily on two adaptations of a T3 genome deleted for its ligase gene and grown on the ligase-defective host. As a preliminary step, the ligase+ T3 wild-type phage (T3^+^) was adapted to the ligase-defective host, yielding T3^+^_E_. The phage ligase gene (*1.3*) was then deleted twice independently from T3^+^_E _to create two stocks for adaptation. The first adaptation (T3Δ1.3A) was carried out on the ligase-defective host for a total of 67 hours. The second adaptation (T3Δ1.3B) was conducted across 4 plate passages and then 45 hours of liquid transfer; plate passages were necessary at first because the fitness of the initial phage was negative and could not be maintained by liquid serial transfer. Each line is subscripted 0 to denote the initial phage isolate immediately following introduction of the deletion, and subscripted E to indicate the 'evolved' or end phage. All assays with T3Δ1.3A_E _used the endpoint population; all assays with T3Δ1.3B_E _used an isolate from the endpoint population. No significance is attributed to the difference in these approaches. The main difference applies to polymorphisms in the evolved population, and any changes remaining polymorphic at the endpoint are likely of too weak a benefit to be detectable as compensatory by our test.

Additional adaptations, mostly short-term, were carried out using the same protocol as above: (i) the ligase-T3 (T3Δ1.3B_0_) was adapted to the ligase-normal host (BL21); (ii) a T3 whose ligase gene was replaced with that of T7 was adapted to the ligase-defective host; (iii) T7^+ ^was deleted for its ligase gene and adapted briefly to the ligase-defective host [this adaptation parallels the prior T7 adaptations reported in [7]].

Fitness is represented as doublings of the phage titer per hour and represents an absolute measure of exponential growth that is independent of generation time. Fitness was assayed under the same conditions as for adaptation except that the phage to cell ratio was kept low in fitness assays. Exponential growth rate was determined with titers from two time points typically separated by at least an hour. Samples to measure titer were treated with chloroform before plating. The titer for the initial time point was taken after 20–60 min of phage growth, to ensure the attainment of a stable 'age of infection' distribution in the culture (enabling a legitimate estimate of an exponential growth rate). The time at which a stable age of infection distribution is 'reached' depends on the average lysis time and on the synchrony of both adsorption and lysis time. We did not measure these factors directly but relied on an empirical determination that growth was approximately exponential (by comparing titers across multiple, consecutive time points). Minimally, two fitness assays per isolate were performed.

### Molecular methods

PCR products or genomic DNA were used as templates for DNA sequencing. Sequences were obtained with an ABI3100 machine using BigDye v. 3.0 for the reactions; sequence profiles were analyzed with DNAStar software.

The deletions of T3 ligase, T7 ligase, and an insertion of T7 ligase were each carried out with plasmid-based, site directed mutagenesis as done previously for T7 [7]. Using overlapping PCR, the desired deletion/insert was created with segments of T3 DNA that flanked the desired insert site. This PCR fragment was cloned into a pUC plasmid, verified by sequencing, and transformed into either host, BL21 or IJ434. Phage T3^+^_E _(adapted to the ligase-defective host) was plated on the plasmid-bearing host to allow recombination with the plasmid. Under the best of conditions, recombinants constitute only 5% of the individual isolates from a plaque, so it was necessary to screen several secondary plaques to be confident of finding a recombinant. Identification of a recombinant thus involved 1–2 additional growth steps: (i) a phage suspension from the plasmid-bearing host was plated onto a ligase-normal host to obtain isolated plaques; (ii) plaques from step (i) were screened for the ligase deletion (or for the transgenic ligase gene). To identify isolates with a deletion of the ligase gene, plaques from step (i) were replica-stabbed onto both a ligase-normal host (BL21) and a ligase-defective host (IJ434). Recombinants lacking ligase were identified from their failure to grow on the ligase-defective host; the stab onto BL21 thus became the stock for the isolate. Phages that had replaced their T3 ligase gene with the T7 ligase gene were identified by PCR after step (i) using T7-specific primers.

To create a phage as a recombinant between two parental phages, the two genomes were digested with a restriction enzyme with a unique site in the genome. Complementary fragments from the two parents were ligated and transfected into IJ1126 (also ligase-normal) to obtain plaques.

### Assaying compensatory changes

As our initial stock of T3^+ ^was not obviously adapted to the ligase-defective host or to other features of the growth conditions used in this study, the wild-type phage T3^+ ^was adapted to the ligase-defective host for 40 hours; growth conditions were the same as used for the deletion phages (described above). Several differences from the published T3 sequence were present by the completion of this pre-adaptation (Table 2; some of those changes may have been present prior to the pre-adaptation). Of course, additional changes might have accumulated had the pre-adaptation been carried out longer, so it cannot be assumed that changes evolving after ligase was deleted were strictly compensatory for the deletion.

We employed a recombination-based method to distinguish strictly compensatory changes from changes that could have evolved even if the ligase gene had been retained. A phage adapted to the absence of its ligase gene (such as T3Δ1.3A_E _or T3Δ1.3B_E_), was allowed to recombine with the parental phage (T3^+^_E_, carrying its ligase gene). A spectrum of recombinant genomes was created by growing the two phage types on a plate with a region of high multiplicity of infection (MOI), thus generating high levels of coinfection (two or more phages infecting the same cell). Recombination among coinfecting genomes is known to involve several breakpoints on average, at least in T7. Furthermore, repeated rounds of coinfection during outgrowth on the plate will produce additional generations of recombinant phages.

The recombinant population was then adapted by serial transfer on IJ434, rapidly resolving the now polymorphic mutations from the adaptation. Since the ligase deletion proved to be intrinsically deleterious, genomes carrying the ligase gene had the highest fitness and ultimately prevailed in this competition. In turn, any mutations also beneficial in a phage background carrying the ligase gene (and thus not strictly compensatory) would accumulate in the ligase genome that ultimately dominates this adaptation; they were identified in the sequence of the final, recombinant population. To determine the consensus genome sequence, the population was sequenced over only those regions known to differ between the initial and evolved genomes, since those were the only changes of interest.

This test is most powerful when the effect of a mutation in the wild-type background is strong. Mutations with weak effect may be influenced by linked mutations or remain polymorphic (polymorphisms are scored as non-compensatory). Therefore, all T3 recombinant adaptations were carried out for at least 10 hours, although a T7 recombinant adaptation was conducted for 6.5 hrs. Furthermore, the T3 lines were initiated with a population of recombinants, grown for 5 hours, then reseeded with recombinant phages to more fully resolve the two classes of mutations.

The recombination test of compensatory evolution can be conducted with evolved populations or with an isolate from the evolved population, but whichever is used should match the template used for sequencing. T3Δ1.3A_E _was sequenced from the evolved population and this population was used for the recombination test. T3Δ1.3B_E _was sequenced from an isolate, and it was used for the recombination test.

Although the focus of the present study is T3, comparison to the original T7 study [7] is an important part of the significance of our results. A 10 hr adaptation of an independent T7Δ1.3 line created for the present study acquired a change in tail gene A, and it was specifically shown to be compensatory by the recombination test.

## Authors' contributions

WRH contributed to the design, made all constructs and carried out the analyses for the T3 A line, conducted the T7 work and helped write the paper. RS made constructs for and did the sequencing for the B line. JJB created and adapted the T3B line, did all fitness assays for the B line, and helped write the paper.

## Supplementary Material

Additional file 1 **Changes observed in the T3Δ1.3 A_E _adaptation and recombination test of compensatory evolution**. Details and status of evolutionary changes observed in the T3Δ1.3 A_E _line, along with known functions of their gene locations. Click here for file

Additional file 2 **Changes observed in the T3Δ1.3 B_E _adaptation and recombination test of compensatory evolution**. Details and status of evolutionary changes observed in the T3Δ1.3 B_E _line, along with known functions of their gene locations. Click here for file
