# Supplementary material for: Compensatory evolution for a gene deletion is not limited to its immediate functional network
Source: BMC Evol Biol. 2009 May 16;9:106. doi: 10.1186/1471-2148-9-106 (PMC2696425; doi:10.1186/1471-2148-9-106)
Supplement: Additional file 1 — Changes observed in the T3Δ1.3 AE adaptation and recombination test of compensatory evolution. Details and status of evolutionary changes observed in the T3Δ1.3 AE line, along with known functions of their gene locations. [file 1471-2148-9-106-S1.pdf]

Additional file 1.

Table 3: Changes observed in the T3Δ1.3A_E adaptation and recombination test of compensatory evolution.

Bases affected (GenBank# AJ318471)	change	Element	Function	T3A_E	R	1st detected (hrs)
2405	G->A G204S	*0.7*	protein kinase	+(2/2)	(0/5)	
5659-6041	deleted and insertion[1]	*ϕ1.05p*, *1.05*, *ϕ1.1*	promoter, ?, promoter	+(1/2)	-	0
6595-7635	engineered deletion	1.3	ligase	+(2/2)	-	
7766-7784	deleted	*1.5*	?	+(2/2)	-	10
7807	C->T R2C	*1.6*	?	+(2/2)	-	
8894	G->A G3S	*2.5*	ssDNA binding	+(1/2)	-	20
9773	T->G F63C	*3*	endonuclease	+(1/1)	-	0
10464	A->G N143S	*3.5*	lysozyme	+(1p/2)	*	
10733	G->A D22N	*4*A	primase	+(2/2)	-	20
12210	T->C L514S	*4*A	primase	+(1/1)	-**	
"	T->C L452S	*4*B	helicase	+(1/1)	-**	
"	T->C W41R	*4.2*	?	+(1/1)	-**	
14186	G->A A382T	*5*	DNA polymerase	(2/2)	+	
17150	G->A	RNAse III *6.5*		+(2/2)	-	40
17493	A->C K4Q	6.7	adsorption	(1/1)	(1/2)	
22531	C->T A36V	11	tail A	+(2/2)	-**	0
24664	A->G Q545R	12	tail B	+(2/2)	-	20
32835	G->A A2T	17	tail fiber	+(1/1)	-	0
33039	T->C Y70H	17	"	+(1/1)	-	0
37606	G->A L4L	*19.5*	?	+(1/1)	+/-	
5665-6006	deleted	*1.05*, *ϕ1.1*	?, promoter	-(1/2)	-	0
6551	C->T	RNAse III *1.3*		+/-(1/2)	-	
11447	G->A V260M	*4*A	primase	+/-(1p/1)	-	
"	G->A V198M	*4B*	helicase	+/-(1p/1)	-	
15321	T->C W52R	*5.3*	?	+/-(1/2)	-**	
15430	A->G H88R	*5.3*	?	+/-(1p/2)	-**	

In the "T3A_E" and "R" columns, a ratio in parentheses indicates the number of isolates in which the mutation was observed over the number of isolates sequenced, whereas a + or – sign indicates the presence or absence of the change in a consensus sequence of the population; +/- indicates multiple peaks in the sequence file. Mutations that were polymorphic in or absent from the consensus of T3Δ1.3A_E are listed under the bold line. Column 'R' is for the recombinant test of compensatory evolution; a "-" entry indicates that the change did not ascend in the test and thus was strictly compensatory except for the engineered ligase deletion. Insertion[1] is a duplication of T3 nucleotides 6515-6551 inserted just after base 5659; this

insertion contains part of the promoter and RNaseIII site upstream of 1.3 and should restore functionality of the ϕ1.05 promoter.

ϕ – promoter; ? – function unknown; * = the mutation listed was absent in the recombinant, but a new mutation in the same codon had replaced it, so its status is ambiguous and is not considered strictly compensatory; -** = sequence was obtained from a mix of 10 isolates from the population; p = multiple sequence peaks;
