# Supplementary material for: Compensatory evolution for a gene deletion is not limited to its immediate functional network
Source: BMC Evol Biol. 2009 May 16;9:106. doi: 10.1186/1471-2148-9-106 (PMC2696425; doi:10.1186/1471-2148-9-106)
Supplement: Additional file 2 — Changes observed in the T3Δ1.3 BE adaptation and recombination test of compensatory evolution. Details and status of evolutionary changes observed in the T3Δ1.3 BE line, along with known functions of their gene locations. [file 1471-2148-9-106-S2.pdf]

Additional file 2

Table 4: Changes observed in the T3Δ1.3B_E adaptation and recombination test of compensatory evolution.

Bases affected (GenBank AJ318471)	Change	Element	Function	T3B_E	R
5781	T->C F22S	*1.05*	?	+	-
6595-7635	engineered deletion	1.3	ligase	+	-
7719	G->A W2X	*1.5*	?	+	-
8807	A->G T46A	*2*	inhibit host RNAP	+	-
9745	C->A P54T	*3*	endonuclease	+	-
10307	A->G K91E	*3.5*	lysozyme	+	-
11211	C->T A181V	*4*A	primase	+	-
"	C->T A119V	*4*B	helicase	+	-
13037	G->A	IG (*5*)	Ribosomal binding site	+	-
15145	A->G K701K	*5*	DNA polymerase	+	-
15265	G->A S33N	*5.3*	?	+	-
16737	A->C T201P	*6*	exonuclease	+	-
17495	A->C K4N	*6.7*	adsorption	+	*
21390	T->A V167D	*10*A	major capsid	+	-
"	T->A V167D	*10*B	minor capsid	+	-
32319	C->A R1173S	*16*	internal core	$+^2$	-
33186	G->A D119N	*17*	tail fiber	+	-
36424	C->T Y278Y	*19*	DNA packaging	+	-
"	C->T T63M	*19.2*	?	+	-

T3Δ1.3B_E changes were determined in the sequence of an isolate. The recombination test was started with a mix of this isolate and with the initial, pre-adapted $T3^+_E$ population; all sequences from the recombination test are based on consensus of the adapted, recombinant population. $+^2$ = chage was present in initial deletion isolate, T3Δ1.3B_0; IG – intergenic change; ? = function unknown; * = the mutation listed was absent in the recombinant, but a new mutation in the same codon had replaced it, so its status is ambiguous and is not considered strictly compensatory. Otherwise as in Additional file 1.
